# Acidity-Controlled Conducting Polymer Films for Organic Thermoelectric Devices with Horizontal and Vertical Architectures

**DOI:** 10.1038/srep33795

**Published:** 2016-09-26

**Authors:** Woongki Lee, Myeonghun Song, Soohyung Park, Sungho Nam, Jooyeok Seo, Hwajeong Kim, Youngkyoo Kim

**Affiliations:** 1 Organic Nanoelectronics Laboratory, Department of Chemical Engineering, School of Applied Chemical Engineering, Kyungpook National University, Daegu 41566, Republic of Korea; 2 Advanced Composites Materials Technical Center, Toray Advanced Materials Korea Inc., Gumi 39389, Gyeongbuk, Republic of Korea; 3 Center for Plastic Electronics, Department of Physics, Blackett Laboratory, Imperial College London, London SW7 2AZ, United Kingdom; 4 Department of Physics, Division of Mathematical, Physical and Life Sciences, University of Oxford, Oxford OX1 3PD, United Kingdom; 5 Priority Research Center, Research Institute of Advanced Energy Technology, Kyungpook National University, Daegu 41566, Republic of Korea

## Abstract

Organic thermoelectric devices (OTEDs) are recognized one of the next generation energy conversion platforms because of their huge potentials for securing electricity continuously from even tiny heat sources in our daily life. The advantage of OTEDs can be attributable to the design freedom in device shapes and the low-cost fabrication by employing solution coating processes at low temperatures. As one of the major OTE materials to date, poly(3,4-ethylenedioxythiophene):poly(styrene sulfonate) (PEDOT:PSS) has been used, but no study has been yet carried out on its acidity control even though the acidic components in OTEDs can seriously affect the device performance upon operation. Here we demonstrate that the addition of aniline (a weak base) can control the acidity of PEDOT:PSS and enhance the performance of OTEDs. In particular, the vertical OTEDs with aniline-doped PEDOT:PSS films (active area = 1.0 cm^2^) could continuously generate electricity (0.06 nW) even at low temperatures (<38 °C) when they were mounted on a desk lamp (power = 24 W).

Thermoelectric devices (TEDs) have been gradually attracting our interest because a variety of heat sources exist in our daily life^1^,^2^,^3^. Some of the TEDs with inorganic materials (ITEDs) have been already commercialized even though their efficiency is still far behind the reasonable values compared with other energy conversion devices^4^,^5^,^6^,^7^,^8^. The efficiency of TEDs is typically represented by the figure of merit, 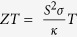, a dimensionless number representing the efficiency, where ***S***, ***σ***, ***κ***, and ***T*** are Seebeck coefficient (thermoelectric voltage per temperature), electrical conductivity, thermal conductivity, and temperature, respectively. Here ***S***^2^***σ*** is also called a power factor, which is a measure of the power efficiency from heat to electricity^9^,^10^,^11^.

To date, the highest ZT values (1.7~2.4) have been achieved from bismuth chalcogenides including bismuth telluride (Bi_2_Te_3_)^12^,^13^,^14^. However, such ITEDs have fundamental demerits owing to the poor mechanical flexibility (bendability) and typically high-temperature processes of inorganic materials, which may limit the wide-spreading of ITED modules^15^,^16^. In this regard, organic TEDs (OTEDs) have attracted keen interest because of their potentials for low-cost flexible plastic TED modules in the coming flexible electronics era^17^,^18^,^19^,^20^,^21^,^22^,^23^,^24^,^25^. Of various organic materials used in OTEDs, conducting polymers have been widely investigated due to relatively high electrical conductivity and good film-forming properties^19^,^20^,^21^,^22^,^23^,^24^,^25^.

However, as most conducting polymers are typically prepared by chemically doping corresponding conjugated polymers with acidic dopants such as sulfonic acid derivatives, perchloric acids, etc., their solutions and films possess inevitably excess acidic dopants leading to a strong acidity^26^,^27^,^28^,^29^. In due course, the strongly acidic states of conducting polymers have a bad influence on the performance and stability of OTEDs because the constituent electrodes directly contact the conducting polymer films^30^,^31^. In order to overcome such acidity issues, the addition of strong base materials has been reported for organic solar cell applications in our previous reports^32^,^33^. However, less attention has been so far paid to the acidity control of conducting polymers in the case of OTEDs, even though the strong acidity issue may be critical to attain the device stability in practical applications. In addition, horizontal device structures have been most widely studied to date due to easy fabrication process, even though they have demerits in practical applications in terms of easy attachment on the surface of heat sources.

In this work, we attempted to control the acidity of conducting polymers by employing a weak base material because its mild acidity can make less adverse effects (de-doping and covalent bond scission) compared to strong bases. As the weak base, aniline was used to modify poly(3,4-ethylenedioxythiophene): poly(styrene sulfonate) (PEDOT:PSS) that is one of the typical conducting polymers studied in the field of OTEDs. The aniline addition dramatically reduced the acidity of PEDOT:PSS solutions and enhanced the thermoelectric performances of OTEDs with horizontal and vertical device architectures. The vertical OTEDs with the aniline-doped PEDOT:PSS films, which are easy for practical applications, exhibited continuous generation of electricity even at low temperatures (<38 °C).

## Reaction and Device Geometry

**Figure 1:**
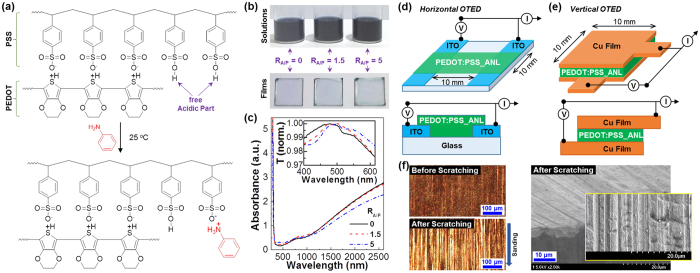
(**a**) Scheme for the aniline (ANL) doping to the free sulfonic acid groups in the PSS units of PEDOT:PSS. (**b**) Solutions and films of PEDOT:PSS according to the amount of aniline (R_A/P_ = 0, 1.5, and 5). (**c**) Optical absorption spectra of films according to the amount of aniline (R_A/P_ = 0, 1.5, and 5) (inset: normalized transmittance). (**d**) Device structures for horizontal (planar) OTEDs which are fabricated with the ITO-glass substrates. (**e**) Device structures for vertical (single stacked) OTEDs which are fabricated with the Cu-foil substrates.

In order to control the acidity of PEDOT:PSS, aniline (ANL) was added to the PEDOT:PSS solutions by varying the molar ratio (R_A/P_) of ANL to the free acidic part (PSS) of PEDOT:PSS. As shown in Fig. 1a, the aniline molecules react with the sulfonic acid groups, which remain freely in the PSS part without involving the doping reaction to the PEDOT parts, by acid-base reactions in solutions. As a result, the free sulfonic acid groups are neutralized with aniline via the proton shift to the aniline from the sulfonic acid groups. Because aniline has a weak yellowish color, the solution color seems to be unchanged even after reaction with a small amount of aniline (see Fig. 1b). However, the film color was slightly changed from bluish to greenish one with increasing the aniline content, which reflects the direct influence of aniline addition as supported from the optical absorption spectra (see Fig. 1c). As shown in Fig. 1d,e, two different types of device geometry (architecture) were introduced in order to examine the thermoelectric performance of the aniline-doped PEDOT:PSS (PEDOT:PSS_ANL) films. The horizontal (planar) geometry is effective in terms of fabrication process because OTEDs can be easily made by just coating the PEDOT:PSS_ANL films on the pre-patterned electrodes (Fig. 1d) but it has a demerit in practical applications because of difficulty in separate mounting of electrodes on either hot or cool region of heat sources. In contrast, the vertical (stacked) geometry is practical for real applications even though OTEDs should be fabricated by stacking the PEDOT:PSS_ANL films in between bottom and top electrodes (Fig. 1e).

## Acidity, Work Function and Morphology

**Figure 2:**
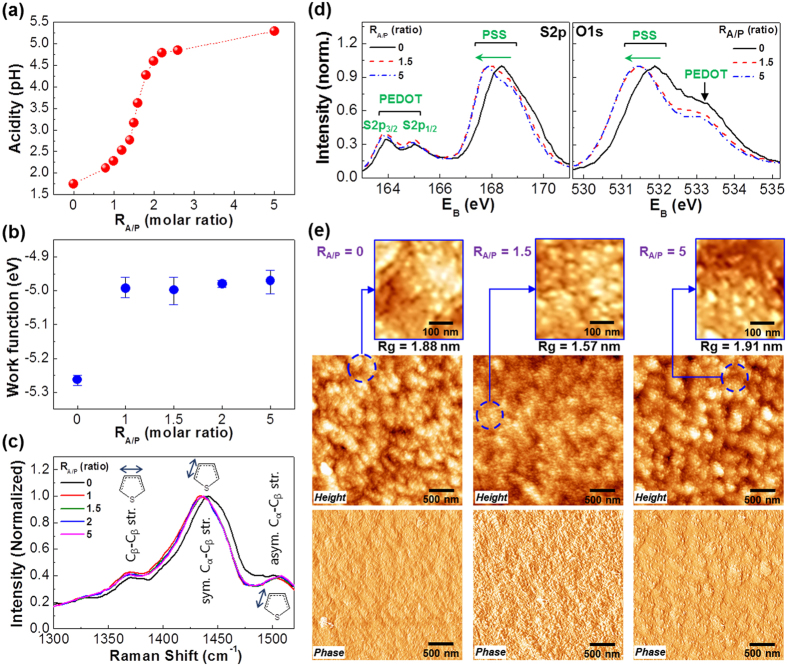
(**a**) Acidity of the PEDOT:PSS solutions according to the aniline ratio (R_A/P_). (**b**) Work functions of the PEDOT:PSS_ANL films according to the aniline ratio (R_A/P_). (**c**) Raman spectra of the PEDOT:PSS_ANL films according to the aniline ratio (R_A/P_): C_β_-C_β_ stretching vibration at ca. 1370 cm^−1^, symmetric C_α_-C_β_ stretching vibration at ca. 1440 cm^−1^, and asymmetric C_α_-C_β_ stretching vibration at ca. 1500 cm^−1^. (**d**) XPS spectra of the PEDOT:PSS_ANL films according to the aniline ratio (R_A/P_): (left) S2p, (right) O1s. (**e**) AFM images (top: height mode, bottom: phase mode) for the PEDOT:PSS_ANL films according to the aniline ratio (R_A/P_): The small images on top were taken from the parts marked with the dotted circles in the height mode images. The root-mean-square roughness (Rg) is given on each image.

As shown in Fig. 2a, the acidity of PEDOT:PSS (aqueous solution) was gradually changed from pH = 1.74 to pH = 6.1 as the aniline content increased. A slow pH increase was measured up to R_A/P_ = 1.4, followed by the swift change in pH value between R_A/P_ = 1.4 and R_A/P_ = 2.2. After R_A/P_ = 2.2, the increasing trend was quite slowed down leading to pH = 5.29 at R_A/P_ = 5. These results inform that the acidity of PEDOT:PSS can be effectively controlled with aniline. Interestingly, the work function of films was noticeably affected by the addition of aniline (see Fig. 2b). When the same amount of aniline to the PSS monomeric unit with free sulfonic acid group (R_A/P_ = 1.0) was added, the work function was noticeably shifted from −5.2 eV to −5.0 eV. However, further addition of aniline did not almost change the work function, which indicates that the electronic properties of PEDOT:PSS are initially (R_A/P_ = 1.0) influenced but insensitive to the presence of more aniline even though the acidity of solutions was greatly affected as discussed in Fig. 2a. This trend is also supported by the Raman spectra on the vibration of thiophene rings in the PEDOT units (Fig. 2c), which deliver clear shift to the low wavenumber direction at R_A/P_ = 1.0 compared to the pristine PEDOT:PSS film (R_A/P_ = 0) but no further shift was made with additional aniline ratios. In particular, it is considered that more π-conjugations through the PEDOT chains could be made by the addition of aniline because the Raman peak (symmetric C_*α*_-C_*β*_ stretching vibration) at ca. 1440 cm^−1^ (benzenoid structure) was shifted to ca. 1415 cm^−1^ (quinoid structure)^34^,^35^,^36^,^37^,^38^.

Next, X-ray photoelectron spectroscopy (XPS) measurements were carried out to investigate the influence of aniline addition on the PEDOT:PSS films. As shown in Fig. 2d, the S2p peak for the PSS part (168.4 eV) was shifted toward the low binding energy direction by the addition of aniline (R_A/P_ = 1.5) but almost no further shift was made even by adding more aniline (R_A/P_ = 5.0). This result informs that the binding energy between sulfur and oxygen atoms in the PSS part became weak owing to the doping reaction of aniline to the sulfonic acid groups of PSS^39^,^40^,^41^. Similarly, the O1s peak for the PSS part (531.9 eV) was shifted toward the lower energy direction by ca. 531.5 eV^37^. As expected, the intensity of the N1s peak was increased with the amount of aniline (see Fig. S1), indicating the presence of aniline molecules doped to the PSS part (note that the present XPS result is not quantitative but qualitative so that the exact amount of doped aniline cannot be obtained). As observed from the atomic force microscope (AFM) images in Figs 2e and S2 the film surface became relatively smoother by the addition of aniline (R_A/P_ = 1.5) but turned rougher again by further addition of aniline (R_A/P_ = 5.0). However, the fine nanoparticles (<40 nm) were measured for all samples (see the enlarged images on top in Fig. 2e), which means that the aniline addition could not alter the intrinsic size of the PEDOT:PSS nanoparticles but could control the aggregation morphology of those basic nanoparticles. The trend of surface roughness can be explained by the role of aniline that might break the aggregates of individual (intrinsic) PEDOT:PSS nanoparticles at lower aniline content (R_A/P_ = 1.5) but could lead to another types of aggregations at higher aniline contents (R_A/P_ = 5) because excess aniline can act as a poor solvent against water in the solutions.

## OTEDs with Planar Geometry

**Figure 3:**
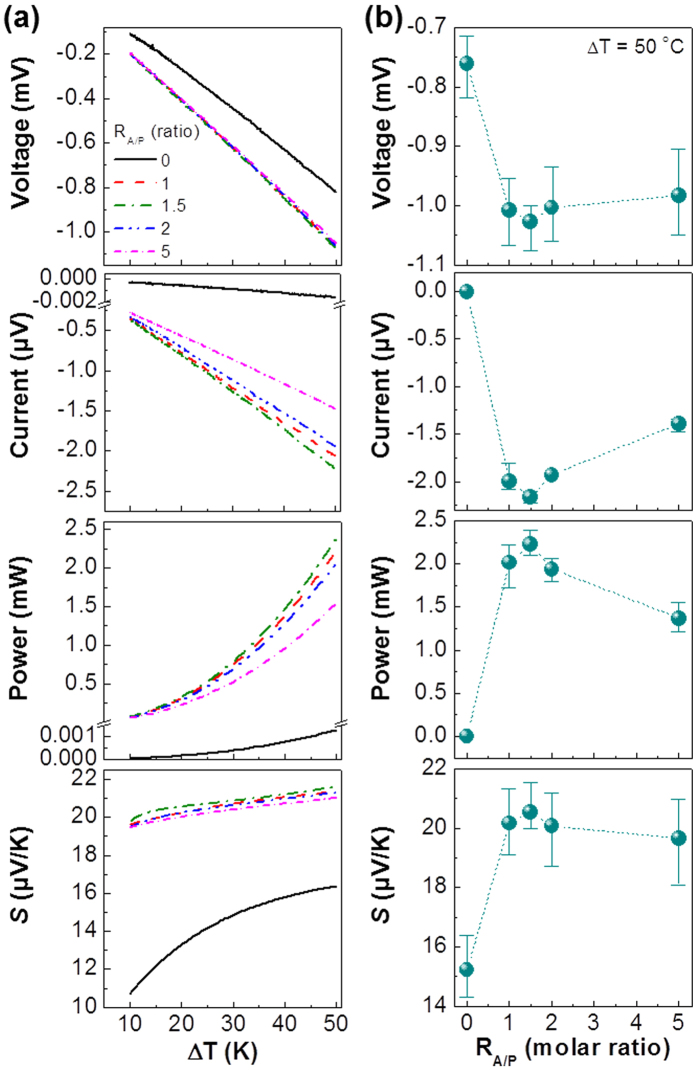
(**a**) Thermoelectric (TE) characteristics (voltage, current, power, and Seebeck coefficient (*S*)) of horizontal (planar) OTEDs with the 70 nm-thick PEDOT:PSS_ANL films according to the aniline ratio (R_A/P_) as a function of temperature difference. (**b**) TE characteristics as a function of R_A/P_ at ΔT = 50 K.

The planar OTEDs with the thin PEDOT:PSS_ANL films (thickness = 70 nm) were fabricated using the patterned ITO-glass substrates (see Fig. 1d). As shown in Fig. 3a (top), the device voltage was gradually increased in the negative direction for all devices as the temperature increased. In particular, the voltage (absolute value) at a fixed temperature difference (ΔT) was higher for the OTEDs with the PEDOT:PSS_ANL films than the control device with the pristine PEDOT:PSS film. The voltage difference at ΔT = 50 °C is summarized in Fig. 3b (top), which delivers the highest voltage at R_A/P_ = 1.5. Similar to the voltage trend, all OTEDs showed the negatively increased current with the temperature in the presence of obvious current difference according to the aniline content (Fig. 3a middle top). The highest (negative) current (−2.224 μA) at ΔT = 50 °C was measured at R_A/P_ = 1.5, which is the same as for the voltage trend, even though only −0.002 μA was measured for the control device. This result indicates that the device performance could be significantly enhanced by the aniline addition. However, more than R_A/P_ = 1.5 led to adverse effect because both voltage and current were gradually decreased with the aniline content. As a consequence, the highest electrical power could be generated at R_A/P_ = 1.5 over the entire temperature range tested in this work (see the power factor trend in Table S1). A particular attention is paid to the electrical power trend that exhibits the higher power generation at the higher temperature, which is partly supported by the trend of Seebeck coefficient for the OTEDs with the PEDOT:PSS_ANL films that are clearly outperforming the control device (see Fig. 3 bottom). The reason for such improvement in device performances can be attributed to the enhanced electrical conductivity of PEDOT:PSS films by the addition of aniline (see Fig. S3). The highest electrical conductivity in the in-plane direction of films was measured at R_A/P_ = 1.5, which can be correlated with the finer morphology leading to the improved contact among the intrinsic PEDOT:PSS nanoparticles with better conjugation lengths at R_A/P_ = 1.5 (see Fig. 2e). Interestingly, the electrical conductivity was slightly more increased with temperature for the PEDOT:PSS_ANL films up to R_A/P_ = 2 but it followed the trend of the pristine PEDOT:PSS at R_A/P_ = 5. This result informs that too much aniline addition has an adverse effect on the electrical properties owing to the morphology change as discussed in Fig. 2e.

**Figure 4:**
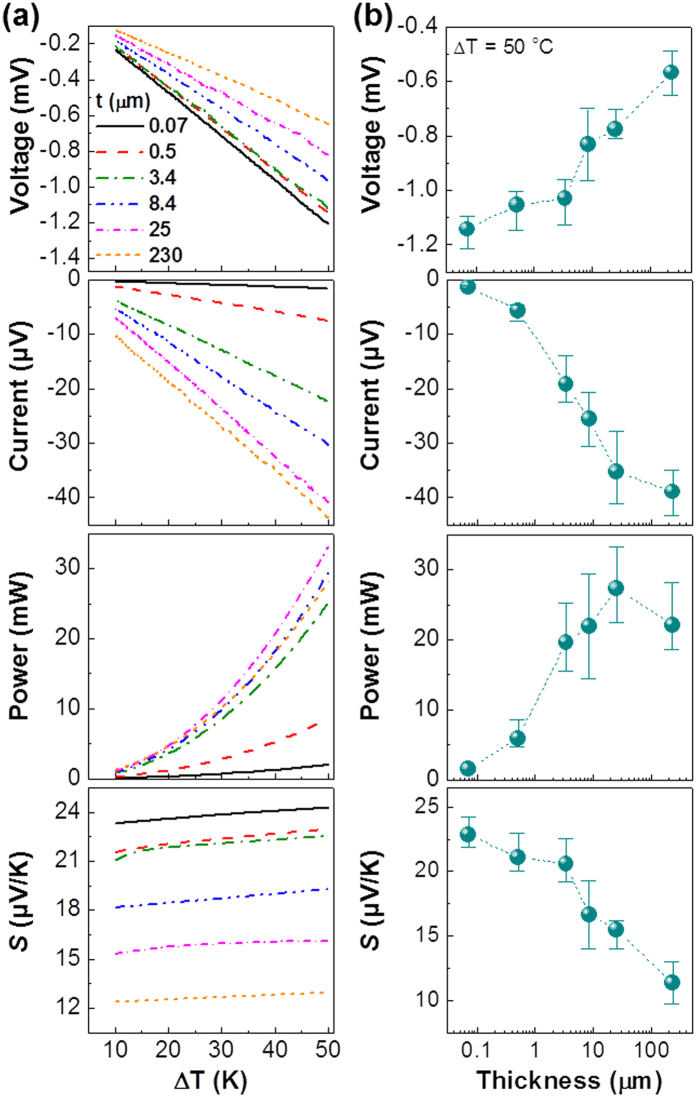
(**a**) Thermoelectric (TE) characteristics (voltage, current, power, and Seebeck coefficient (*S*)) of horizontal (single stacked) OTEDs with the PEDOT:PSS_ANL films (R_A/P_ = 1.5) according to the film thickness (*t*) as a function of temperature difference. (**b**) TE characteristics as a function of film thickness at ΔT = 50 K.

Next, the thickness of the PEDOT:PSS_ANL films was changed by fixing the aniline content at R_A/P_ = 1.5. As shown in Fig. 4a, the device voltage was almost linearly increased to the negative direction with the temperature irrespective of the film thickness. This result supports that the thickness range (from 70 nm to 230 μm) here is appropriate to operate OTEDs with proper thermoelectric functions. As the film thickness increased, the (negative) slope of device voltage became gradually low leading to the decreasing trend in the Seebeck coefficient (see also Fig. 4b). In contrast, the device current was pronouncedly increased from −1.639 μA to −43.30 μA as the film thickness increased from 70 nm to 230 μm. This means that more charges could be generated in the thicker films due to their relatively larger volumes. As a result, according to the bigger influence of device current, the higher electrical power generation was achieved for the thicker films even though the electric power became slow down after 25 μm (see the power factor trend in Table S2).

## OTEDs with Vertical Geometry

**Figure 5:**
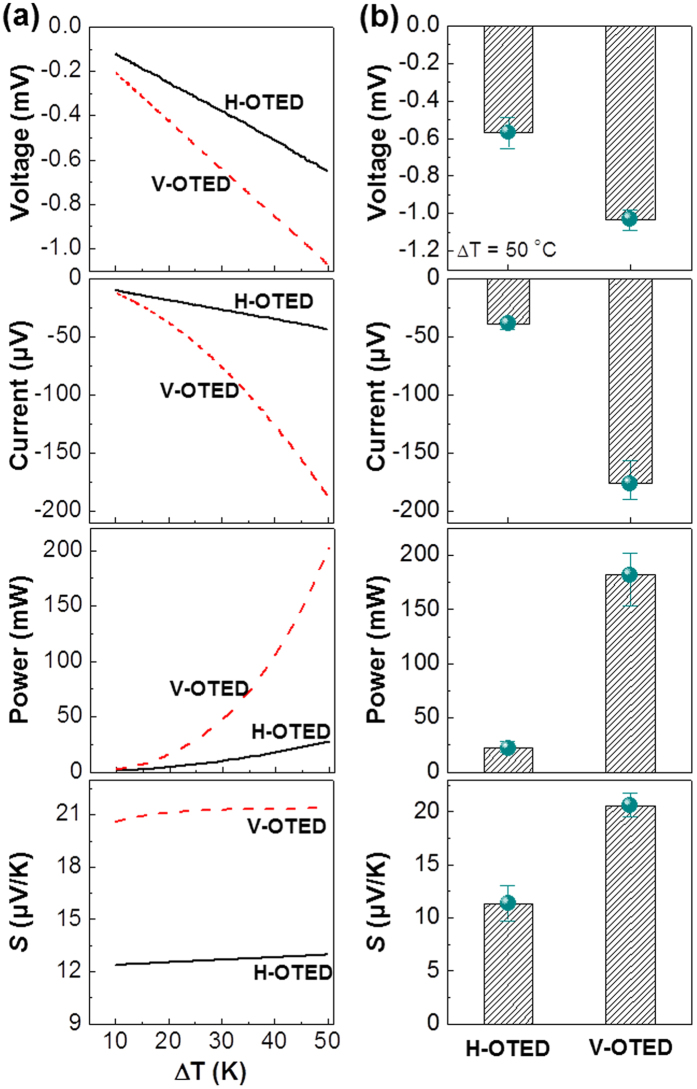
(**a**) Comparison of thermoelectric (TE) characteristics (voltage, current, power, and Seebeck coefficient (*S*)) between horizontal (H-OTED) and vertical (V-OTED) devices with the 230 μm-thick PEDOT:PSS_ANL films (R_A/P_ = 1.5) as a function of temperature difference.(**b**) TE characteristics between the two OTEDs (H-OTED and V-OTED) at ΔT = 50 K.

The thick (230 μm) PEDOT:PSS_ANL films were employed for the fabrication of the OTEDs with the vertical geometry as discussed in Fig. 1e. The planar OTEDs were also fabricated at the same time for exact comparison. As shown in Fig. 5a, the device voltage was almost linearly increased with temperature irrespective of the device geometry. However, the vertical OTED showed higher voltage than the planar OTED over the whole temperature range tested in this work. In particular, the voltage difference between the two OTEDs became much greater at higher temperatures. Similarly, the device current was also increased with temperature but the current gap between the two OTEDs became much more pronounced as the temperature increased. The vertical OTED generated −189.5 μA at ΔT = 50 °C, which is almost 4-fold of the current (−43.30 μA) generated in the planar OTED (see Fig. 5b). As a result, the vertical OTED could produce higher electrical power than the planar OTED. The outstanding performance of the vertical OTED is also supported by the higher Seebeck coefficient that reached 21.37 μV/K for the vertical OTED compared to 13.04 μV/K for the planar OTED at ΔT = 50 °C. The TE parameters are summarized in Table S3, in which the influence of coating method on the device performance is given for the OTEDs with the thin (70 nm) PEDOT:PSS_ANL films for reference.

**Figure 6:**
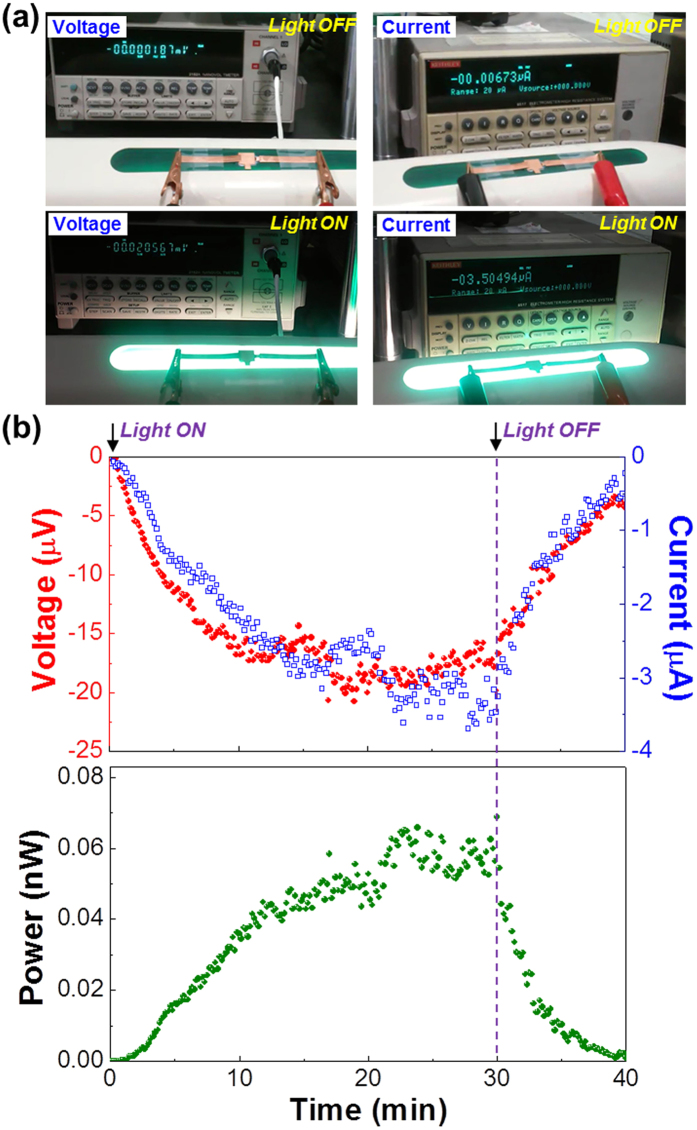
Practical applications of vertical OTEDs with the 230 μm-thick PEDOT:PSS_ANL films (R_A/P_ = 1.5). (**a**) Photographs for vertical OTEDs mounted on a desk lamp and the voltage/current measurement systems before and after the lamp light on. (**b**) Change of thermoelectric characteristics for the vertical OTEDs mounted on a desk lamp after turning on the desk lamp. The active area of the vertical OTEDs was 1.0 cm^2^.

Finally, in order to examine the actual applicability, the vertical OTEDs with the 230 μm-thick PEDOT:PSS_ANL films were attached on the top cover of a desk lamp (see Fig. 6a). When the lamp light was turned on, the device voltage was gradually increased till 10 min. Then the voltage was fluctuated owing to the local temperature variation around the device, followed by saturation due to no more temperature rise at around 20 min. When the lamp was turned off, the voltage was slowly decreased for ca. 20 min because of cooling process in the lamp cover part. The trend of device current was quite similar to that of device voltage in the presence of slightly different fluctuation behavior between 10 min and 30 min. This is why the device current measurement was performed independently after finishing the voltage measurement, which might cause slightly different temperature conditions in the lamp cover part. Accordingly, the electrical power showed the similar rise and decay trend (see the bottom graph in Fig. 6b). This short test strongly supports that the present vertical OTEDs with the PEDOT:PSS_ANL films are convenient for practical applications and can continuously generate electrical power even in indoor environments (see Fig. S4 and video clips).

## Outlook

It has been demonstrated that the acidity of PEDOT:PSS can be effectively controlled by adjusting the amount of aniline (a weak base). The aniline addition led to the enhanced electrical conductivity in the presence of the work function change in the PEDOT:PSS films. As a result, the performance of OTEDs was significantly improved by the addition of aniline and the highest electrical power could be achieved at the aniline content of R_A/P_ = 1.5. The detailed study showed that 25 μm-thick PEDOT:PSS_ANL films delivered the highest electrical power at the optimum aniline content (R_A/P_ = 1.5). In particular, the vertical OTEDs with the PEDOT:PSS_ANL films exhibited superior performances compared to the planar OTEDs. The practical application test disclosed that the vertical OTEDs are easy to mount on any heat sources and can generate electric power continuously even at low temperatures below 38 °C. This result strongly supports that the vertical OTEDs are almost ready for real applications in terms of device operations. However, the stability (lifespan) of OTEDs need to be investigated in the next step. In this regard, it is briefly demonstrated here that the PEDOT:PSS_ANL films are effective to prevent the corrosion of metal electrode because the acidic components in the PEDOT:PSS films were removed (neutralized) by the addition of aniline (see Fig. S5). Hence the lifespan of OTEDs is expected to be greatly improved by the present aniline addition technology.

## Methods

### Aniline doping reaction

PEDOT:PSS solutions (PH500, concentration = 1.0~1.3 wt%, PEDOT:PSS = 1:2.5 by weight) were received from Heraeus (Germany). The unreacted molar ratio of PSS parts was calculated from the weight ratio of PSS to PEDOT in the PEDOT:PSS solutions. To prepare the aniline-doped PEDOT:PSS solutions, aniline (ANL) was first dissloved in dimethyl sulfoxide (DMSO, 250 μl) because it is insoluble in water. Then the ANL/DMSO mixtures were added to the PEDOT:PSS solutions, followed by stirring for well mixing for more than one week at room temperature. The molar ratio (R_A/P_) of ANL to the free acidic part (PSS) of PEDOT:PSS was varied from R_A/P_ = 1 to R_A/P_ = 5 by fixing the amount of DMSO (5 vol%) to the PEDOT:PSS solution.

### Film and device fabrication

To fabricate OTEDs with a horizontal device architecture, indium-tin oxide (ITO)-coated glass substrates (24 mm × 24 mm, sheet resistance = 10 Ω/□) were subject to a photolithography/etching process to make the ITO strip electrodes (10 mm × 24 mm) with a 10 mm gap. After wet cleaning using deionized water, acetone, and isopropyl alcohol (IPA), the patterned ITO-glasses were treated with a UV-Ozone (UVO Cleaner, Ahtech LTS, Republic of Korea, 15 mW/cm^2^) for 20 min in order to remove any organic residues. Next, the aniline-added PEDOT:PSS (PEDOT:PSS_ANL) films were spin-coated or drop-coated from the ANL-added PEDOT:PSS solutions. In the case of spin-coating at 1500 rpm for 60 s, the film thickness was 70 nm. The thick films (70 nm~230 μm) was prepared by drop coating at the fixed R_A/P_ = 1.5. All films after coating were followed by baking at 60 °C for drying. For the fabrication of vertical OTEDs, copper (Cu) foil substrates (thickness = 300 μm) were tailored to make the foil (plate) size of 1 cm × 1 cm and one side of the tailored Cu foils was rubbed with a sandpaper (Grit size = 2000, average particle diameter = 10.3 μm) in order to make surface scraches for better adhesion with the PEDOT:PSS_ANL films. These Cu foil plates were cleaned using acetone and IPA for 30 min and treated with a UV-Ozone for 20 min. One set of two Cu plates was placed on a hot plate at 60 °C and 200 μl of the PEDOT:PSS_ANL solutions (R_A/P_ = 1.5) was dropped on each plate. After drying for ~30 min, the two Cu plates with the PEDOT:PSS_ANL films were sandwiched and dried for 48 h at the same temperature. The active area of the vertical OTEDs was 1.0 cm^2^.

### Measurements

The acidity of solutions was measured using a pH meter (Accumet AB15, Fisher Scientific). The thickness of thin films (<10 μm) was measured using a surface profilometer (Alpha-step 200, Tencor), while an optical microscopy (SV-55, Sometech) was used to measure the thickness of thicker films (>10 μm). The optical absorption spectra of films were measured with a UV-visible spectrometer (Lambda 750, PerkinElmer), while the work function of films was measured using a photoelectron spectrometer (AC-2, Hitachi High Tech, UV intensity = 10.0 nW, counting time = 10 s, anode voltage = 3,040 V, measured from 4.20 to 6.20 eV at atmospheric condition). The Raman spectra of films was measured with a Raman spectrometer (Nicolet Almega XR, Thermo Scientific) by fixing the laser excitation wavelength of 532 nm. The atom environment change before and after aniline doping was analyzed using a X-ray photoelectron spectroscopy (XPS, ESCALAB 250Xi, Thermo Scientific). The surface morphology of films was measured with an atomic force microscope (AFM, Nanoscope IIIa, Digital Instruments). The voltage-temeperature-current characteristics of OTEDs were measured using a home-built TE measurement system equipped with a nanovoltameter (2182A, Keithley) and a high-sensitivity source-measure meter (6517, Keithley) which are connected with a control unit (NI 9211, National Instrument) that enables accurate temperature sensing via a proportional-integral-derivative (PID) program. The application test of devices was performed by mounting the vertical OTEDs with the 230 μm-thick PEDOT:PSS_ANL films on the top cover of a desk lamp (GO-780, G. K. World, 24 W), in which an extended Cu strip was used as a lead electrode for proper attachment of the devices (see Figs 6 and S4).

## Additional Information

**How to cite this article**: Lee, W. *et al*. Acidity-Controlled Conducting Polymer Films for Organic Thermoelectric Devices with Horizontal and Vertical Architectures. *Sci. Rep.* **6**, 33795; doi: 10.1038/srep33795 (2016).

## Supplementary Material

Supplementary Information

Supplementary Video 1

Supplementary Video 2
