# Supplementary material for: Acidity-Controlled Conducting Polymer Films for Organic Thermoelectric Devices with Horizontal and Vertical Architectures
Source: Sci Rep. 2016 Sep 26;6:33795. doi: 10.1038/srep33795 (PMC5035924; doi:10.1038/srep33795)
Supplement: Supplementary Information [file srep33795-s1.pdf]

[Supporting Information]

Acidity-Controlled Conducting Polymer Films for Organic Thermoelectric Devices with Horizontal and Vertical Architectures

Woongki Lee[1], Myeonghun Song[1], Soohyung Park[1,2], Sungho Nam[1,3,4], Jooyeok Seo[1], Hwajeong Kim[1,5,*], and Youngkyoo Kim[1,*]

[1]*Organic Nanoelectronics Laboratory, Department of Chemical Engineering, School of Applied Chemical Engineering, Kyungpook National University, Daegu 702-701, Republic of Korea*

[2]*Advanced Composites Materials Technical Center, Toray Advanced Materials Korea Inc., Gumi 730-400, Gyeongbuk, Republic of Korea*

[3]*Center for Plastic Electronics, Department of Physics, Blackett Laboratory, Imperial College London, London SW7 2AZ, United Kingdom*

[4]*Department of Physics, Division of Mathematical, Physical and Life Sciences, University of Oxford, Oxford OX1 3PD, United Kingdom*

[5]*Priority Research Center, Research Institute of Advanced Energy Technology, Kyungpook National University, Daegu 702-701, Republic of Korea*

*Corresponding Authors : Prof. Y. Kim (ykimm@knu.ac.kr)

Dr. H. Kim (khj217@knu.ac.kr)

Supplementary Table 1. Summary of thermoelectric characteristics for OTEDs with the PEDOT:PSS_ANL films according to the aniline ratio ($R_{A/P}$) at ΔT = 50 K.

$R_{A/P}$ (molar ratio)	0	1	1.5	2	5
Voltage (mV)	-0.819	-1.067	-1.076	-1.060	-1.049
Seebeck Coefficient (μV/K)	16.37	21.33	21.53	21.20	20.97
Current (μA)	-0.002	-2.087	-2.224	-1.945	-1.476
Electrical Conductivity (S/cm)	0.3	279.5	295.2	262.2	201.1
Power (nW)	0.001	2.225	2.393	2.062	1.548
Power Factor ($\mu W/m/K^2$)	0.007	12.72	13.68	11.78	8.845

ΔT = 50 ^{o}C

Supplementary Table 2. Summary of thermoelectric characteristics for OTEDs with the PEDOT:PSS_ANL films ($R_{A/P}$ = 1.5) according to the film thickness at ΔT = 50 K.

Thickness (μm)	0.07	0.5	3.4	8.4	25	230
Voltage (mV)	-1.213	-1.148	-1.126	-0.964	-0.809	-0.652
Seebeck Coefficient (μV/K)	24.26	22.95	22.53	19.28	16.17	13.04
Current (μA)	-1.639	-7.487	-22.38	-30.51	-41.10	-43.30
Power (nW)	1.988	8.591	25.21	29.42	33.23	28.22
Power Factor ($\mu W/m/K^2$)	11.36	6.873	2.966	1.401	0.532	0.049

ΔT = 50 ^{o}C

Supplementary Table 3. Summary of thermoelectric characteristics for OTEDs with the PEDOT:PSS_ANL films ($R_{A/P}$ = 1.5) according to the device geometry (architecture) at ΔT = 50 K. H-OTED and V-OTED denote horizontal and vertical OTEDs, respectively.

	**H-OTED 70 nm Film Spin-Coating**	**H-OTED 70 nm Film Drop-Coating**	**H-OTED 230 µm Film Drop-Coating**	**V-OTED 230 µm Film Drop-Coating**
**Voltage (mV)**	-0.941	-1.213	-0.652	-1.069
**Seebeck Coefficient (µV/K)**	18.82	24.26	13.04	21.37
**Current (µA)**	-2.472	-1.639	-43.30	-189.5
**Electrical Conductivity (S/cm)**	375.3	193.0	2.888	0.004
**Power (nW)**	2.326	1.988	28.22	202.5
**Power Factor (µW/m/K^2)**	13.29	11.36	0.049	0.0002

ΔT = 50 ^{o}C

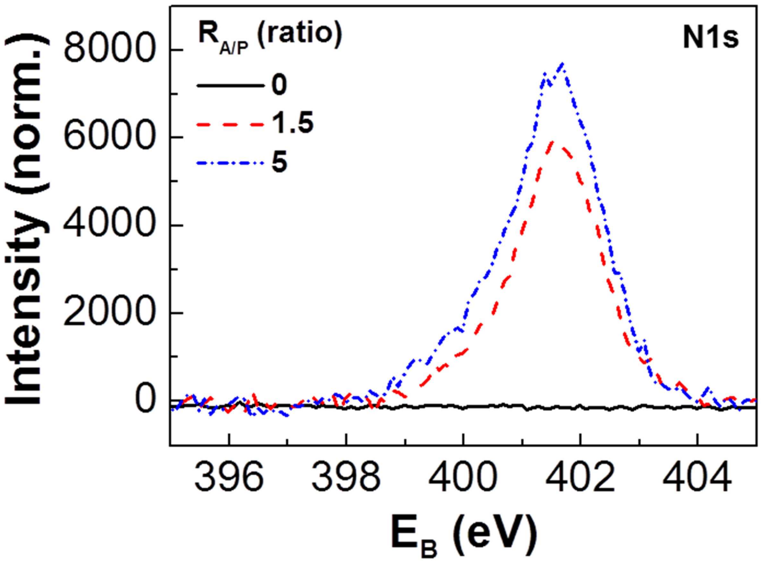

Supplementary Figure 1. N1s XPS spectra of the PEDOT:PSS_ANL films according to the aniline ratio ($R_{A/P}$): The present XPS spectra were normalized for better comparison by setting the intensity of baseline region (<398 eV) to zero, even though XPS cannot generally guarantee the quantitative analysis because it does actually measure the localized surface part (several tens of nanometers in depth), not the entire part, in the films.

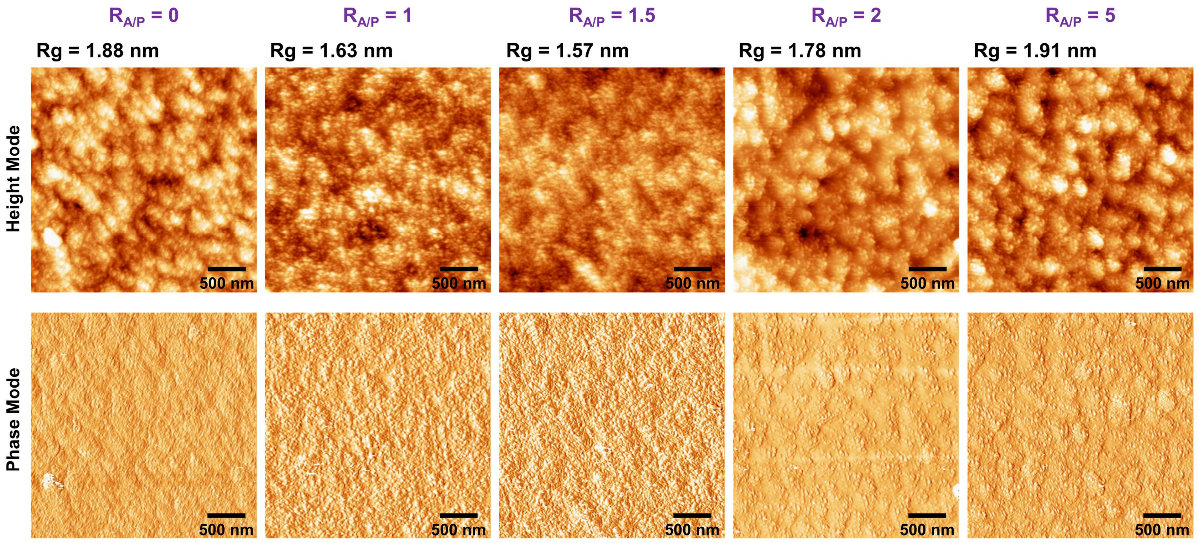

Supplementary Figure 2. AFM images for the PEDOT:PSS_ANL films according to the aniline ratio ($R_{A/P}$): (top) height mode images, (bottom) phase mode images. The root-mean-square roughness (Rg) is given on each image.

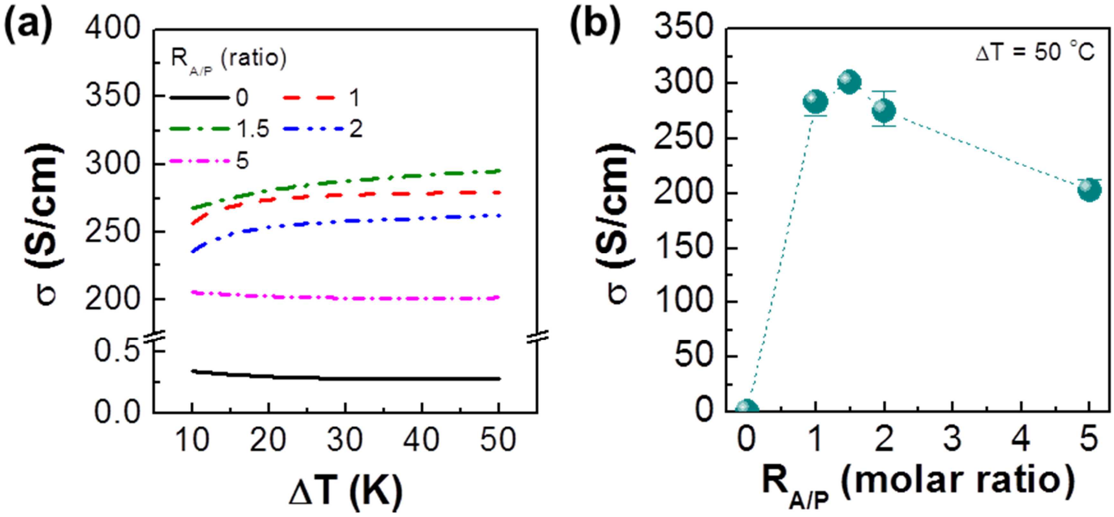

Supplementary Figure 3. Electrical conductivity for the OTEDs with the PEDOT:PSS_ANL films as a function of temperature difference (a) and the aniline ratio ($R_{A/P}$) at ΔT = 50 K.

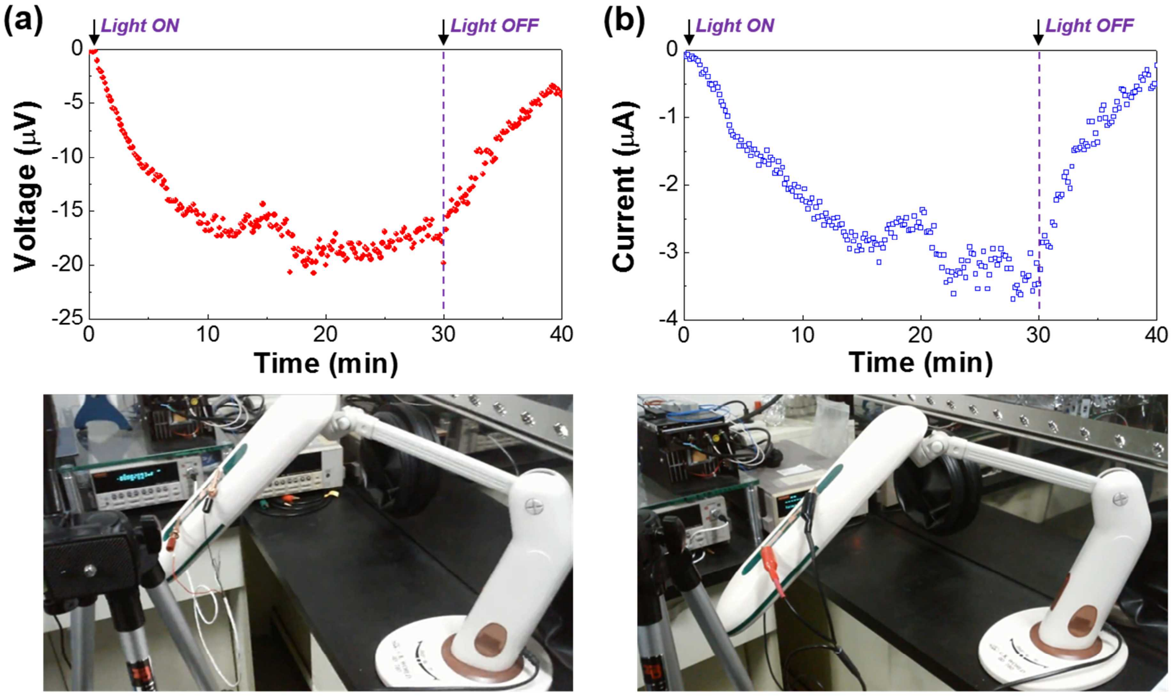

Filename: voltage.avi

Filename: current.avi

Supplementary Figure 4. Practical applications of vertical OTEDs with the 230 μm-thick PEDOT:PSS_ANL films ($R_{A/P}$ = 1.5): (a) Voltage and (b) current changes as a function of time. "Light ON" and "Light OFF" denote the turn on and off time of the desk lamp (24 W) on which the OTEDs are mounted (see photographs and video clips included). The active area of the vertical OTEDs was 1 cm^2.

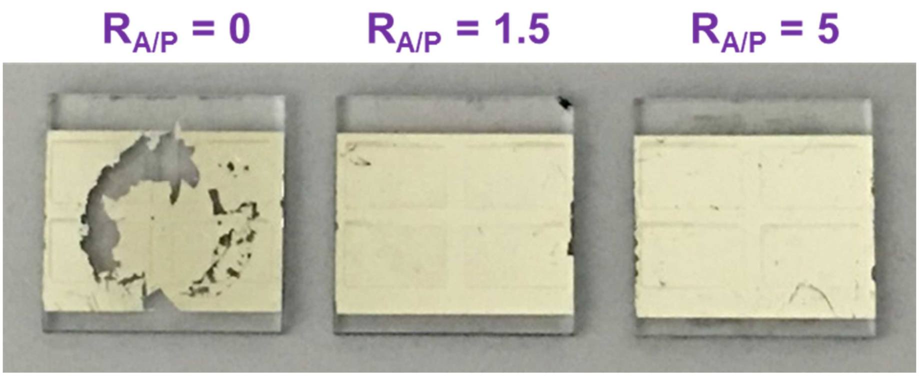

Supplementary Figure 5. Photographs taken for the aluminum (60 nm)-coated glasses on which the PEDOT:PSS solutions prepared with different aniline content ($R_{A/P}$) were dropped and kept for 60 min at room temperature before cleaning with deionized water. The Al electrode was significantly damaged by the drop of the pristine PEDOT:PSS solution (pH = 1.75), whereas almost no damage in the Al electrodes was observed in the case of the aniline-doped PEDOT:PSS solutions (pH = 3.16 for $R_{A/P}$ = 1.5 and pH = 5.29 for $R_{A/P}$ = 5.0).
